# Competency of novice anesthesia residents in performing trans thoracic echocardiography following a structured problem-based hands-on course using a trans thoracic echocardiography simulator versus video-based training: a randomized controlled assessor-blinded trial

**DOI:** 10.1186/s41077-026-00406-1

**Published:** 2026-01-24

**Authors:** Bharat Yalla, Manpreet Kaur, Abhishek Nagarajappa, Rashmi Ramachandran, Vimi Rewari, Thilaka Muthiah, Bikash Ranjan Ray, Arshad Ayub

**Affiliations:** 1 Department of Anaesthesiology, Pain Medicine, and Critical Care,All India Institute of Medical Sciences, New Delhi, India; 2 Department of Anesthesia and Perioperative Medicine, Penn State Milton S Hershey Med Center, Hershey, PA USA; 3 Apollo Simulation Centre chennai, Apollo Hospitals and Apollo Institute of Medical Sciences and Research, Chittoor, India

**Keywords:** Simulation, Trans thoracic echocardiography, Anesthesia, TTE competency score

## Abstract

**Background:**

Trans-thoracic echocardiography (TTE) improves the diagnostic skills of anesthesia trainees during the perioperative period. We compared performance between two educational intervention, simulation-based hands-on training and video-based training as measured by a novel TTE Competency Score (TCS).

**Methods:**

Fifty novice anesthesia residents were randomized into an intervention group-I (received simulation-based training) and a control group-C (received video-based training). Both groups underwent evaluations of their knowledge and skills on live volunteers or ICU patients using TCS right after the training and again six months thereafter. An independent sample t-test was employed to compare TCS between the groups, while a paired t-test was used to assess intragroup TCS at the baseline, after training, and six months post-training.

**Results:**

The two groups had no significant difference in the pre-test assessment scores. Immediately following the training session, Group I exhibited a significantly higher TCS compared to Group C (25.2 ± 2.7 and 22.4 ± 2.5, p = 0.01). Compared to the I-group, the C-group made more attempts to achieve competence. Group I exhibited higher confidence levels both immediately and six months later. After a six-month follow-up, the TCS scores in both groups were lower than the scores recorded immediately.

**Conclusion:**

This study demonstrates that a structured, problem-based simulator course improved immediate TCS and 6-month confidence compared with video-based training. However, a decline in TCS in both groups over time highlights the need for periodic refreshers within six months to improve retention.

**Trial registration:**

CTRI [CTRI/2021/07/034684].

**Supplementary Information:**

The online version contains supplementary material available at 10.1186/s41077-026-00406-1.

## Background

Trans-thoracic echocardiography (TTE) is a non-invasive procedure that can be performed bedside with immediate results. TTE has become a part of the core curriculum of cardiologists and intensivists [1, 2]. With TTE now widely used in operation theatres, emergency departments, and critical care units, it is time to include it in the anesthesia curriculum.

Simulation-based training enables learners to develop clinical and decision-making skills through real-life situational experiences [3]. Previous studies have shown the benefits of simulation-based echocardiography training, most focused on transesophageal echocardiography (TEE) in anesthesia programs rather than TTE in general anesthesia trainees [4–7]. 

Despite calls to include focused TTE in non-cardiology curricula [8–11], there is no consensus on the most effective way to teach image acquisition and interpretation to novice anaesthesia residents. Simulation offers a promising hands-on approach, but evidence comparing structured simulator training to other scalable methods like video-based instruction and evidence on skill retention and real-patient transferability in TTE remains limited.

Previous randomized studies in ultrasound-guided regional anesthesia and in TEE have demonstrated that simulation-based training significantly improves hands-on image acquisition and psychomotor performance compared with video-based or didactic instruction [12, 13]. However, data specific to focused TTE training in anesthesia residents, remain limited.

Various assessment methodologies have been proposed to ensure trainee’s TTE competency [10, 11], but these are very complicated or have been extrapolated from TEE assessment scoring, and there are currently no widely accepted or validated assessment tools specifically designed for evaluating competency in focused TTE among anaesthesia trainees.

We hypothesized that anesthesia trainees who receive structured simulation enhanced TTE training would demonstrate higher TTE Competency Score (TCS) in TTE image acquisition and interpretation, and retention of these skills at 6 months post-training would be higher compared to trainees who receive video-based training alone.

This randomized assessor-blinded trial was designed to compare a structured, problem-based simulator course versus video-based training in novice anaesthesia residents, using a combined knowledge-and-skill assessment TCS and a six-month follow-up to evaluate retention and translation to live patient assessment.

## Materials and methods

This prospective randomized controlled assessor-blinded study trial was conducted in the Department of Anaesthesiology, Pain Medicine and Critical Care, All India Institute of Medical Sciences, New Delhi, India, after obtaining approval from the institutional ethics committee [IECPG-297/28.04.2021] and informed consent and CTRI [CTRI/2021/07/034684]. The current trial is reported according to the CONSORT (Consolidated Standards of Reporting Trials-Simulation Based Research) guidelines [14]. (CONSORT-SBR Checklist-Additional file 1)

Anesthesia residents with a minimum of six months of training in the department were included. Residents who had previously participated in TTE training, those who had already performed TTE in the past, and those who refused to participate were excluded.

All residents were given basic training in normal anatomy and basic views of TTE through a didactic lecture and demonstrated on simulator introducing the TTE simulation model (CAE-VIMEDIX). After this initial basic training, a 10-point questionnaire (Pre-test assessment) about basics was given, and students were assessed regarding their baseline pre-course knowledge about TTE.

Participants were then randomly allotted to two study groups using a computer-generated random table with a sealed opaque envelope technique for allocation concealment. Study Group I (Intervention Group) received hands-on reinforcement training on the TTE simulator (TTE Protocol- Additional file 2 ) on normal views and five pathologic clinical conditions (hypovolemia, poor left ventricular function, poor right ventricular function, regional wall motion abnormalities, cardiac tamponade), that provided guided probe positioning, image optimization feedback, and repetitive deliberate practice over two weeks, with an instructor: learner ratio of 1:3, and the same facilitators ran all sessions, and the control group (Group C) received video sessions/ based training with pre-recorded video modules covering identical content (standard views and the same five pathologies). Video access was self-directed during the two weeks; no hands-on simulator time was provided, and facilitators were available for technical queries only.

The assessment was done using TCS (which includes Knowledge component [multiple-choice questions], and Skill component [image acquisition and pathology identification])(TTE Competency score-Additional file 3), with the assessor interrogating about normal views and 2 pathologies was blinded to the group’s allocation. The endpoint of the assessment was image plane acquisition of 5 standard TTE views, identifying critical features of each pathology.

The assessment was performed using the echocardiography probe of an ultrasound machine (Fujifilm Sonosite, Inc. Bothell, USA). All assessments were performed on live patients and volunteers after obtaining consent.

The primary objective was to assess the TCS of novice anesthesia residents immediately.

The secondary objectives assessed were the time taken for image plane acquisition of each standard TTE view and the diagnosis of a clinical scenario. The number of attempts made to diagnose and to achieve an optimal image plane was noted. Self-assessment of students’ level of confidence after the course was done using a Likert score, and students were assessed for retention of skills after six months using normal/pathological scenarios on the TTE simulator using a TCS.

### Validity evidence (Kane Framework)

The TTE competency score was developed through expert consensus (two from Anesthesiology, one from Cardiac Anesthesia, one from Cardiology, and one Simulation lead), to evaluate foundational transthoracic echocardiography skill. Scoring assumption was ensured by mapping each scored item to standard TTE training objectives used in perioperative and critical care practice. Generalisation was supported by inter-rater reliability during 10 pilot learner examinations (Intraclass correlation coefficient = 0.77, two-way random effects, indicating good agreement). Extrapolation was supported by the use of clinically relevant pathological cases reflecting typical perioperative/ICU presentations. The score was used for formative educational assessment, consistent with the implications level of Kane’s framework, rather than high-stakes certification.

As there are no previous studies available on this score, we conducted a preliminary pilot study with 25 students in each arm (both the intervention and control groups), for a total of 50 students.

Statistical analysis was performed using STATA14.0 (College Station, Texas, USA). Appropriate data analysis was performed based on the data distribution. Intergroup competency was assessed using an independent-sample t-test. TCS at baseline after training and at 6 months intragroup was analyzed using a paired t-test. The number of attempts was analyzed using an independent t-test. Statistical significance was set at *p* < 0.05.

## Results

**Fig. 1:**
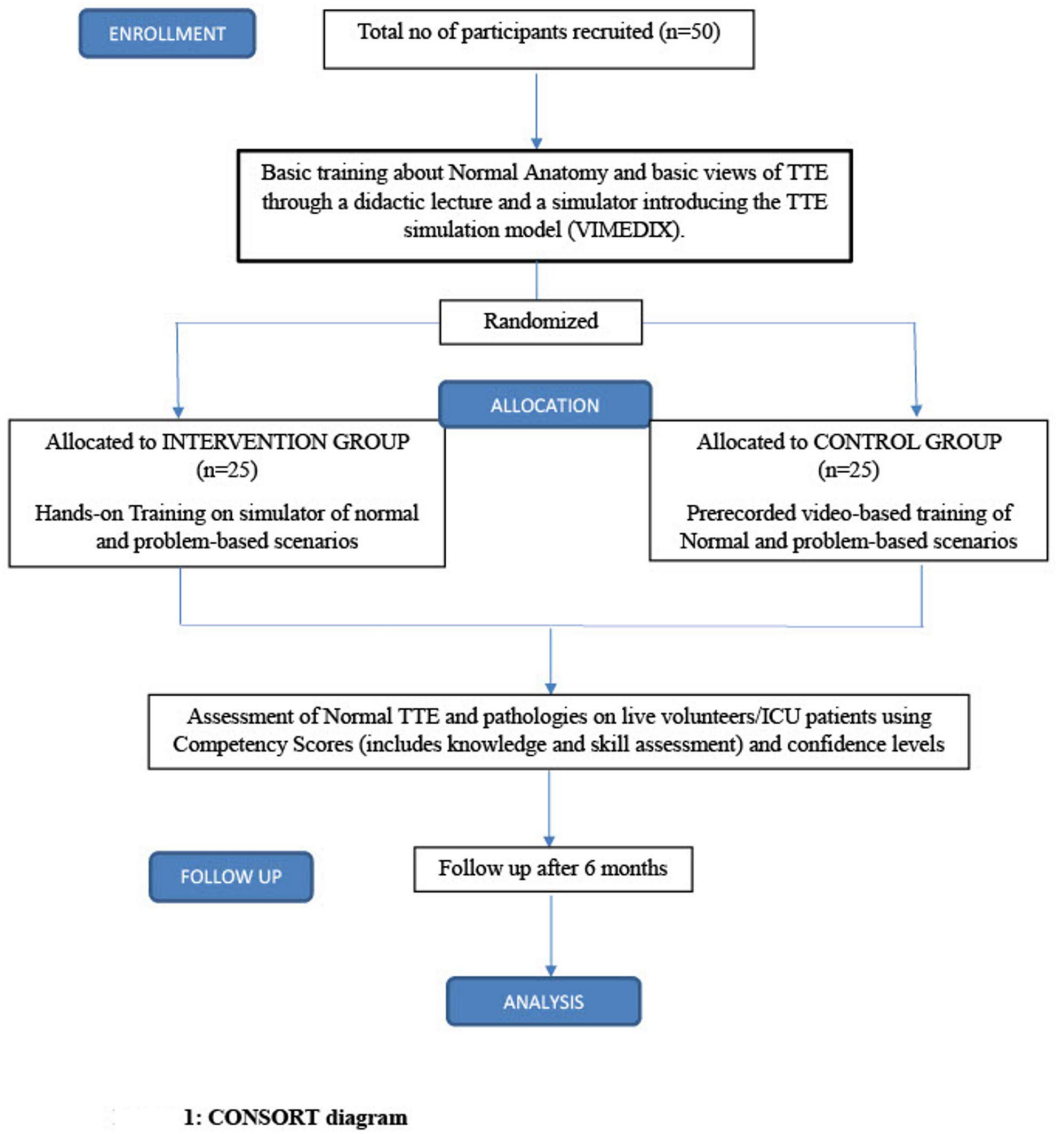
CONSORT (Consolidated Standards of Reporting Trials) diagram

A total of 50 participants were recruited, with 25 participants in each group, for 18 months with a 6-month follow-up. Figure 1 no participants were lost to follow-up. There was no significant difference in the mean age of all the participants in Group I and Group C (25.2 ± 0.6 vs. 25.2 ± 0.5 years), respectively. The gender distribution between the two groups was also statistically similar between the two groups. (Table 1)

**Table 1:** Baseline characteristics

	Intervention group (I) (*n* = 25)	Control group (c) (*n* = 25)
Age (years)	25.2 ± 0.6	25.2 ± 0.5
Female	11 (44%)	12 (48%)
Male	14 (56%)	13 (52%)
Pre-test Assessment score (mean ± SD)	6.8 ± 0.17	6.8 ± 0.18

*mean ± SD * mean ± Standard Deviation, *CI * Confidence Interval

### Pre-test assessment

The pre-test assessment scores for the Intervention and Control groups showed no notable difference. (Table 2)

**Table 2:** Competency scores assessed immediately, total time and time taken for attaining each view, no of attempts, time taken to diagnose pathological scenarios, and confidence level

	Intervention group (I) (*n* = 25)	Control group (c) (*n* = 25)	*p* value
Competency score assessed immediately	25.28 ± 2.79	22.44 ± 2.58	0.01*
Knowledge Assessment score	8.32 ± 0.14	8.16 ± 0.12	0.41
Total time (sec)	286.6 ± 119.1	301.8 ± 118.3	0.574
Time for PLAX (sec)	45.3 ± 39.4	49.2 ± 36.7	0.64
Time for PSAX (sec)	30.2 ± 27.0	27.4 ± 21.8	0.46
Time for A4C (sec)	73.2 ± 35.9	69.2 ± 39.9	0.76
Time for S4C (sec)	51.5 ± 32.6	72.0 ± 44.5	0.06
Time for SIVC (sec)	86.3 ± 49.1	84.0 ± 49.7	0.96
No of attempts	1.64 ± 0.7	2.20 ± 0.5	0.03*
Time taken to diagnose 1st pathological scenario (sec)	175.5 ± 116.4	189.4 ± 113.7	0.55
Time taken to diagnose 2nd pathological scenario (sec)	124.9 ± 111.3	217.1 ± 151.0	0.01*
Confidence level immediately	3.92 ± (0.64)	3.64 ± (0.63)	0.128
Confidence level after 6 months	3.64 ± (0.12)	3.08 ± (0.11)	0.002

*mean ± SD * mean ± Standard Deviation, *CI* Confidence Interval

*Statistically significant

### Knowledge assessment immediately

There was no significant difference in the Knowledge Assessment score done as a part of the TCS between the two groups Table 2.

### TTE Competency Score (TCS)

Immediately following the training session, the intervention group demonstrated significantly higher TCS than the control group.(25.2 ± 2.7 vs. 22.4 ± 2.5, *p* = 0.01). 

### Total time taken for image plane acquisition

No statistical significant difference was observed in the total time required for image plane acquisition, as well as the time taken for acquiring each standard view, including the Parasternal long axis view (PLAX), Parasternal short axis (PSAX), Apical four-chamber (A4C), Subcostal four-chamber (S4C), and Subcostal inferior vena cava (SIVC) views, between the two groups Table 2.

### Number of attempts

The Control group made a significantly greater number of attempts to achieve competency (Competency score > 27/31) compared to the intervention group.(2.20 ± 0.5 vs. 1.64 ± 0.7, *p* = 0.03) respectively. Table 2.

### Time taken to diagnose pathological scenarios

There was no significant difference in the time taken to diagnose the first pathological scenario between two groups but there was a significant difference in the time taken to diagnose the second pathological scenario Table 2.

### Participants’ level of confidence

Confidence level (immediately) was higher in participants trained by using a simulator (Intervention group) than in the control group, but the results were not statistically significant (3.92 ± 0.6 vs. 3.64 ± 0.6, *p* = 0.128), respectively. Confidence level (after 6 months) was higher in the intervention group and was statistically significant (3.64 ± 0.12 vs. 3.08 ± 0.11, *p* = 0.002). Table 2.

### Retention of skills

TCS assessed after 6 months of follow-up in the intervention group were lower than scores assessed immediately and were statistically significant (22.68 ± 2.56 vs. 25.28 ± 2.79, 95% CI 1.8–3.3, *p* = 0.001), with a 10.1% decline in scores. TCS assessed after 6 months of follow-up in the control group were lower than the scores assessed immediately, but were not statistically significant (21.32 ± 2.05 vs. 22.44 ± 2.5, 95% CI 0.1–2.4, *p* = 0.086), with a 4.82% decline in scores. Table 3.

**Table 3:** Competency scores assessed immediately and after 6 months

Competency Scores	*n*	Mean ± SD	95% CI		*p*-value
			Lower	Upper	
Intervention group at 0m	25	25.28 ± 2.7	1.8	3.3	0.01*
Intervention group at 6m	25	22.68 ± 2.5			
Control group at 0m	25	22.44 ± 2.5	0.7	2.4	0.08
Control group at 6m	25	21.32 ± 2.0			

*mean ± SD*  mean ± Standard Deviation, *CI * Confidence Interval

*Statistically significant

## Discussion

This randomized controlled pilot study evaluated whether a structured, problem-based hands-on transthoracic echocardiography (TTE) curriculum using a high-fidelity simulator improves TCS among novice anesthesia residents compared with a video-based instructional approach. Our findings indicate that, while knowledge acquisition was comparable between the groups, residents who participated in simulation-based training exhibited significantly higher TCS. They required fewer attempts and demonstrated enhanced diagnostic performance in a complex pathological scenario. These findings indicate that simulation meaningfully enhances the practical, psychomotor, and interpretative elements of TTE learning beyond what can be achieved through passive video-based instruction alone [12, 13, 15]. 

The primary objective was the TCS, which evaluated both knowledge and skills. The knowledge aspect of the competency score was found to be comparable between the two groups, likely due to the comprehensive standardized 2-hour didactic training provided to both groups. This suggests that to develop competency, the incorporation of extensive, high-quality standardized didactic instruction is crucial when designing a curriculum. The utility of combined teaching and training sessions is further highlighted by studies like Jonathan Kline et al. [8] where a blended curriculum that integrates online didactic instruction with supervised practical training has demonstrated that the majority of participants attained basic proficiency in focused transthoracic echocardiography (TTE) following the intervention. Their results support the concept that deliberate practice within a standardized training framework enhances learner performance. Similarly, our study reinforces that the mode and structure of training play a key role in skill acquisition and retention, underscoring the importance of guided experiential learning rather than unstructured exposure.

Our analysis revealed a notable difference in the TCS immediately following the training session. The intervention group, which received training using a simulator, demonstrated superior TCS compared to the control group, although the absolute difference of 2.8 points may appear numerically small, it represents approximately 9% of the maximum score. Within this assessment framework, this margin corresponds to the difference between reliably acquiring all required TTE views independently versus requiring corrective guidance or repeated attempts. Therefore, the observed difference reflects a clinically meaningful improvement in functional image acquisition competency, rather than a trivial numerical change.

Our results are consistent with earlier studies highlighting the benefits of simulation-based echocardiography training. Neelankavil et al. [6] demonstrated that simulator-based TTE training improves early technical performance in anaesthesia residents. However, most previous work has evaluated learners solely within simulator environments, limiting understanding of real-world transferability. In contrast, our study assessed residents on live volunteers and ICU patients, demonstrating that simulation-gained skills translate directly into clinical performance where acoustic window variability and real-time clinical interpretation challenges exist.

Hands-on training using the simulator not only improved the basic skills in achieving standard TTE views, but also made them proficient in diagnosing pathological scenarios. Our study assessed the participants using two pathological scenarios. There was no statistically significant difference in the time to make the diagnosis for the first pathology, while the diagnosis was made earlier in the second scenario in the intervention group.

The initial pathological scenario (pathology1- cardiac tamponade) was designed to be straightforward, to increase confidence. This was followed by a more intricate pathological scenario (Pathology 2, including Left Ventricular Dysfunction, Right Ventricular dysfunction, and Regional Wall Motion Abnormality). The outcomes demonstrated that residents who underwent simulator training performed better than those in the control group.

There is limited literature available on TTE utility for teaching and training but more on TEE. Gwenael Prat et al. [16] and Andreas Bloch et al. [17] demonstrated that incorporating simulator sessions into the standard curriculum improved the learning curve for TEE hemodynamic assessment in ICU and cardiac pathologies in ICU, respectively.

Although integrating TTE simulation into a structured training program improved technical and practical skills in non-cardiology residents, it did not lead to corresponding improvements in diagnostic or interpretative performance [18]. In contrast, our research demonstrated an improvement in diagnostic capabilities, as evidenced by the higher TCS achieved by the simulator group when diagnosing complex pathologies.

In contrast to previous studies, such as Hempel et al. [7], which assessed participants at a single time point following simulation training, the key contribution of our study is the assessment of skill retention at six months. Both groups experienced a decline in competency scores over time, but the simulation-trained group retained higher long-term confidence and maintained relatively better proficiency. This aligns with known patterns of procedural skill decay, where performance deteriorates without deliberate reinforcement [19]. Ultrasound-based image acquisition skills, in particular, require repeated practice to maintain psychomotor memory and pattern recognition capabilities [20]. The observed decline in both groups highlights the necessity for structured interval reinforcement curricula to ensure sustained competency in focused TTE. Implementing a repeated simulation session at a three-month interval could potentially enhance skill retention.

The confidence levels were statistically equivalent in both groups immediately following the initial session, which was attributable to high-quality, extensive structured didactic training. However, residents trained through simulation exhibited significantly higher confidence after a 6-month interval, indicating the long-term impact of multimodality training and simulation. A study by Damp et al. [21] on TEE in cardiology residents demonstrated that trainees reported increased confidence after simulation-based training, as measured using a confidence-integrated scoring system.

Another important finding relates to the lack of a validated, widely accepted TTE assessment tool for novice perioperative trainees. Existing frameworks, including TEE scoring systems and cardiology-based evaluation rubrics, may not align with the foundational competencies needed for perioperative decision-making The TCS used in this study was developed with expert input across anesthesiology, cardiology, and simulation education, and reflects core bedside TTE skills; however, it is not yet externally validated. This highlights an ongoing gap in the field: despite increasing integration of focused TTE into anesthesia and critical care training, standardized competency assessment has not kept pace with curriculum expansion.

### Strengths and limitations

There are limited simulation studies of TTE in anesthesiology residents, and in addition to standard views of TTE, this study incorporated pathological scenarios and analyzed the retention of skills.

It was hypothesized that learners possessed comparable baseline knowledge; however, potential variations in their clinical practice might have introduced bias. Additionally, selection bias could arise from the characteristics of volunteers or ICU patients utilized for evaluating the trainees, potentially influencing skill assessment. This potential bias was mitigated by the evaluator, who verified the basic views prior to assessing the learner.

The TCS has not been previously validated in external settings; however, this study provides initial validity evidence based on Kane’s framework, including expert-derived scoring criteria (two from anesthesiology, one from Cardiac Anesthesia, one from cardiology, and one simulation lead), good inter-rater reliability, alignment with recognized TTE training objectives, and use for formative skill assessment rather than high-stakes decisions.

However, the skill assessment component of the TCS is subjective, as is the case with most skill assessment scores [10–16] which constitutes another limitation of our study.

To control for confounding factors, both intervention and control groups were trained during the same period; participants had similar background characteristics, and volunteers/patients used for evaluation of skills were pre-scanned by the trainer to assess the difficulty in obtaining normal views.

In the context of Miller’s pyramid for evaluating clinical competence in medical education, the skill of image acquisition is classified under the “Shows” or “Does” levels of competence. Nevertheless, the training competencies may not correspond precisely with these levels.

The sample size was limited, and high-fidelity manikins are expensive and may not be accessible at all institutions.

Another limitation of our study is the potential for differences in instructor interaction between the groups. Although both groups received equal total training time, participants in the simulator-based training arm may have had more opportunities for individualised feedback and facilitator engagement during hands-on sessions. This difference in instructional support may have influenced group performance.

## Conclusion

This study demonstrates that simulation-based TTE training is more effective than video-based learning for developing core image acquisition and pathology recognition skills in novice anesthesia residents and that these gains translate to bedside clinical performance. The inclusion of a six-month follow-up provides important insight into skill retention and reinforces the need for periodic refresher training to maintain competency. The study also highlights the need for further development and validation of standardized, practical assessment tools to support competency-based TTE education in anesthesiology.

## Supplementary Information

Supplementary Material 1.

Supplementary Material 2.

Supplementary Material 3.

## Data Availability

The datasets used and/or analysed during the current study are available from the corresponding author on reasonable request.

## Abbreviations

Group I: Intervention group
Group C: Control group
TTE: Tans-Thoracic Echocardiography
TEE: Trans Esophageal Echocardiography
TCS: TTE Competency Scoring
ICC: Intraclass Correlation Coefficient
CCE: Critical Care Echocardiography
CTRI: Clinical Trial Registry of India
CONSORT: Consolidated Standards of Reporting Trials
mean ± SD: mean ± Standard Deviation
CI: Confidence Interval
