# Supplementary material for: Competency of novice anesthesia residents in performing trans thoracic echocardiography following a structured problem-based hands-on course using a trans thoracic echocardiography simulator versus video-based training: a randomized controlled assessor-blinded trial
Source: Adv Simul (Lond). 2026 Jan 24;11:11. doi: 10.1186/s41077-026-00406-1 (PMC12911289; doi:10.1186/s41077-026-00406-1)
Supplement: Supplementary file 1 — Supplementary Material 1. [file 41077_2026_406_MOESM1_ESM.docx]

**TRANSTHORACIC ECHO Protocol**

CAE VIMEDIX Ultrasound Simulator was used for TTE. It is a high-fidelity simulator with advanced learning features and facilitates cardiac, lung, and abdominal ultrasonography through one common platform. It provides volumetric scanning in real-time with detailed views of the normal heart and advanced pathologies.

Five standard TTE views taught and assessed were:

1. Parastemal long-axis view (PLAX)

2. Parastemal short-axis view (PSAX)

3. Apical view

4. Sub costal four-chamber view

5. Sub costal inferior vena cava (IVC) view

**Details of Views**

l. **Parasternal long-axis view (PLAX):**

The echo probe is placed in the left second intercostal space with the marker facing the right shoulder of the patient (11 o'clock). To optimise this view, the Mitral valve, and atrial valve are placed in the center of the image, make the AV cusps appear symmetric (rotate probe), make the IVS and posterior LV was appeared horizontal (change interspace) and one should not be able to visualize the LV apex (rotate probe).


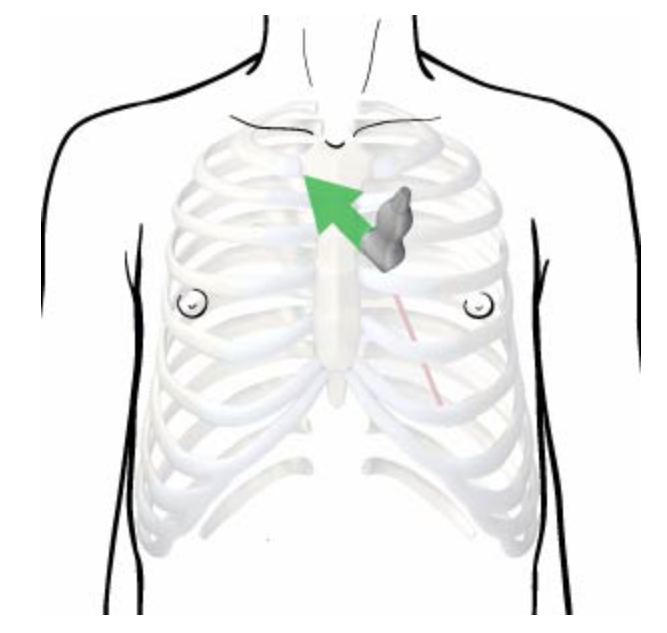

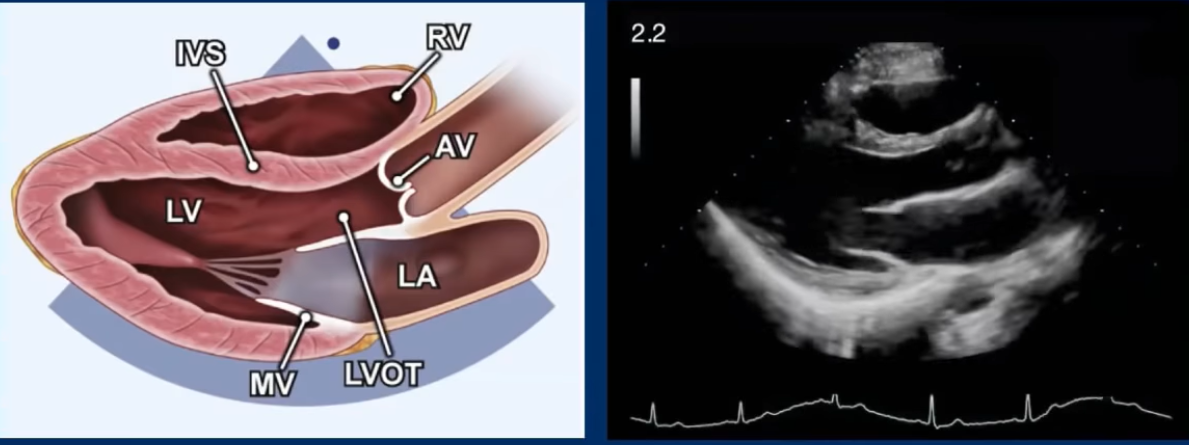


**2. Parasternal short-axis view (PSAX):**

The echo probe is rotated 90 degrees clockwise from the Parasternal LAX view so that the marker on the probe points towards the left shoulder of the patient (2 o'clock). The view can be optimised by making the LV appear round in shape and the RV crescent-shaped


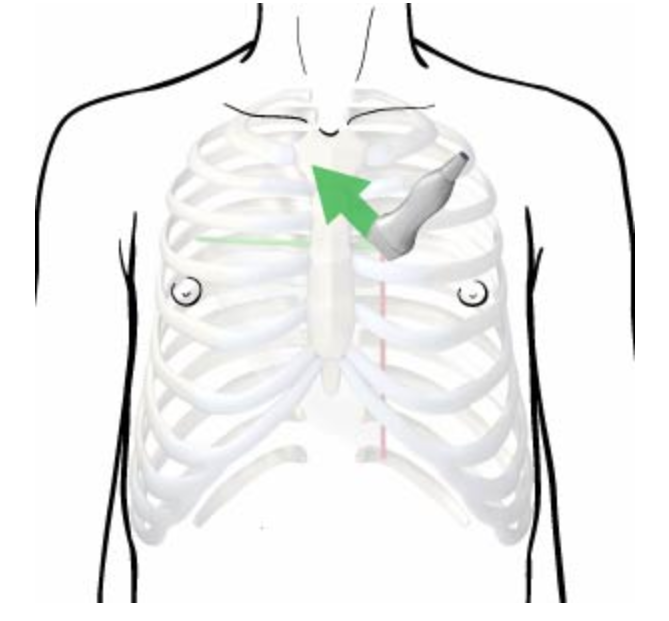

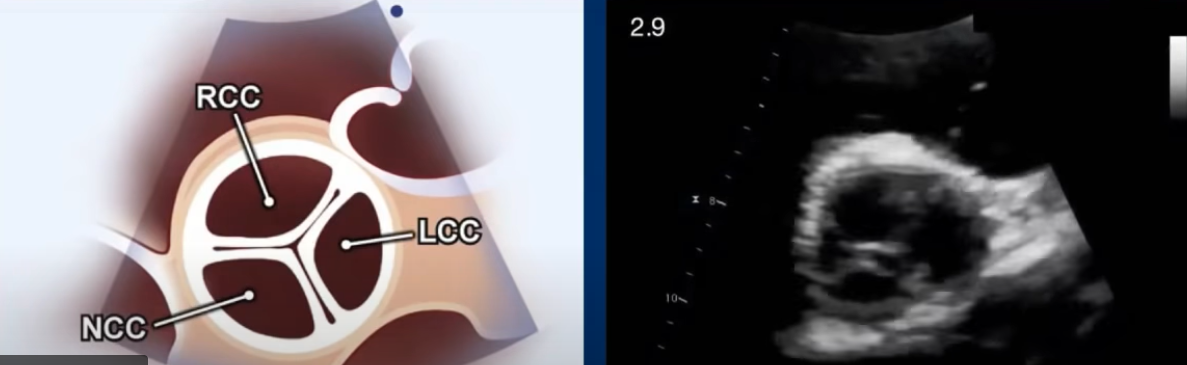

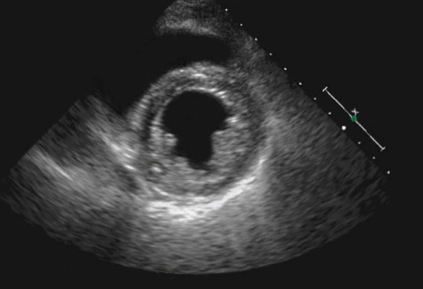


**3. Apical view:**

The probe is placed in the 4th or 5th intercostal space in the midclavicular line or at the point of apical pulsation with the index marker pointing towards the left (3 o'clock). For optimal view ensure all chambers are visible and the interventricular septum appears vertical and in the center of the image.


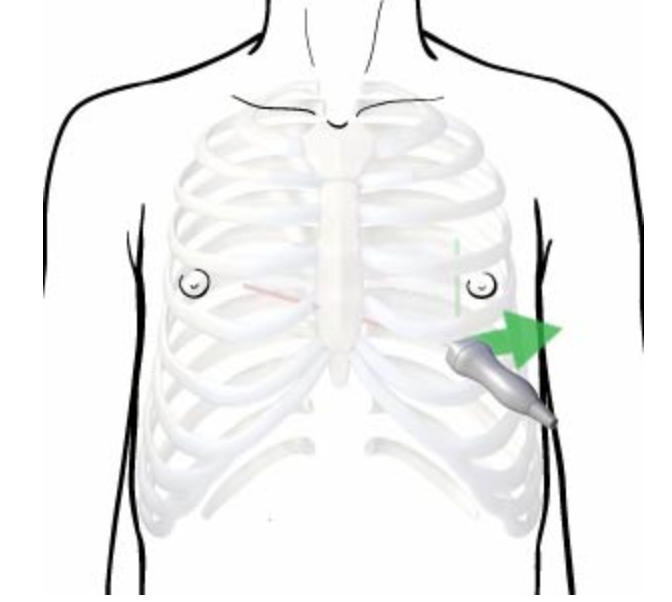

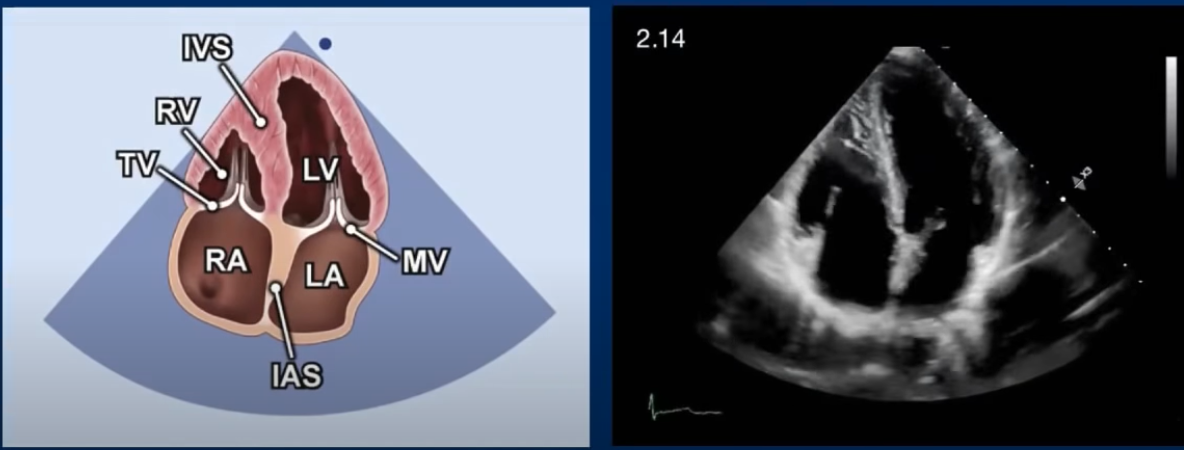


**4. Sub costal four-chamber view**

The probe is placed in the subcostal area with the marker facing left and is tilted upwards to look at the heart (3 o'clock). For optimal view, all four cardiac chambers should be visible and the entire LV including the apex are seen.


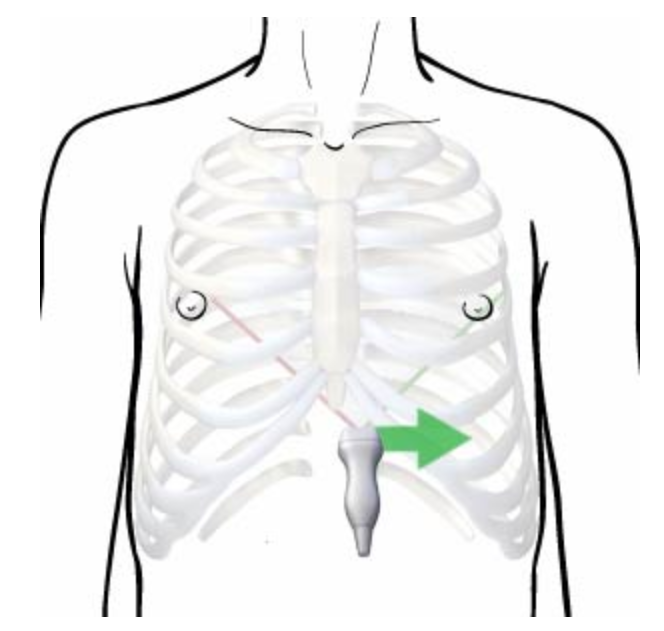

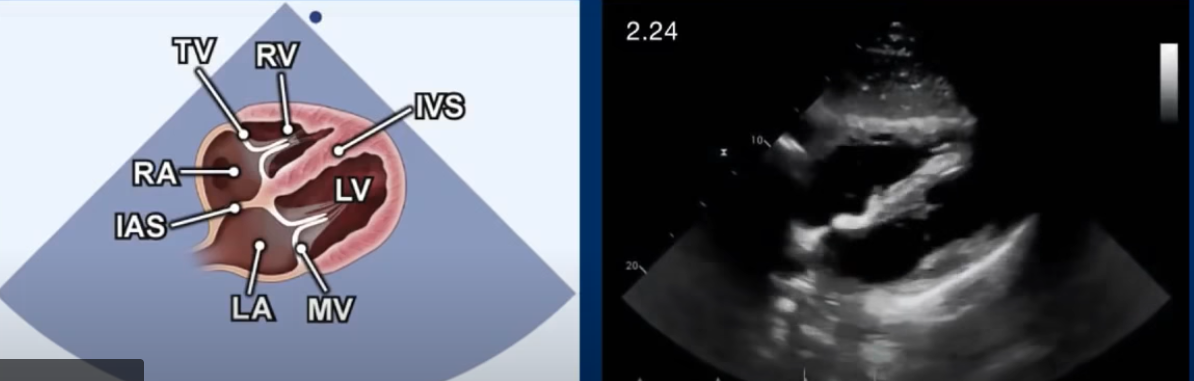


**5.Sub costal inferior vena cava (IVC) view:**

It is obtained by rotating the probe 90 degrees counterclockwise from the subcostal 4C view. For an optimal view, the junction of the IVC and Rt atrium should be visible and IVC should appear horizontal.


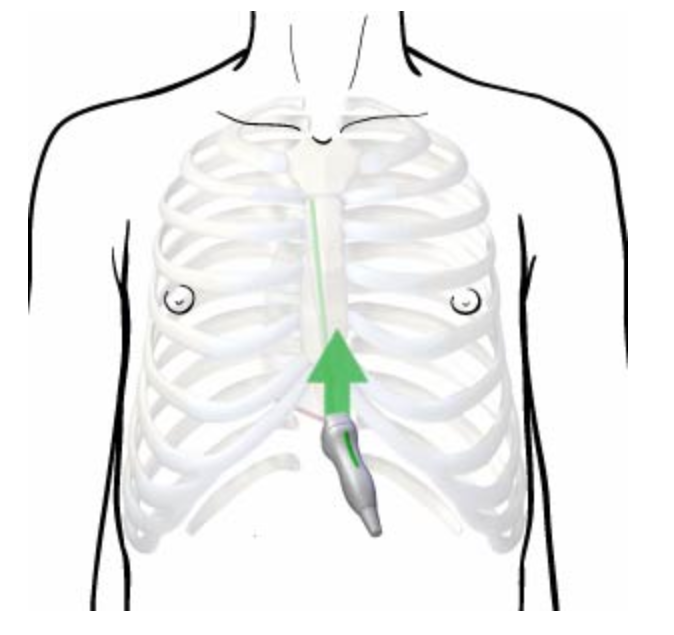

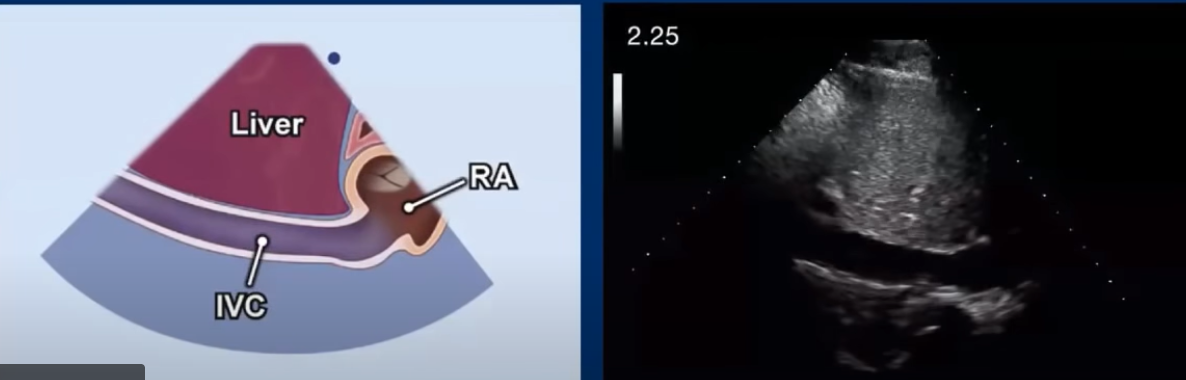


Critical features of Pathological CLINICAL SCENARIOS to be used:

1. **Hypovolemia:**

- PSAX view showing “kissing papillary muscles”
- LVEDA of less than 10 cm2
- IVC size and collapsibility: The IVC size and collapsibility are assessed in the subcostal IVC view. Size ≤ 2.1 cm; collapses >50% during sniff = RAP 0–5 mm Hg indicates hypovolemia and fluid responsiveness

1. **Poor LV function:** Look for the cavity size, mitral valve excursion by Eyeballing, EPSS (E point septal separation), Ejection fraction using M-Mode, and mid-ventricular size during systole by eyeballing over left ventricular function.
2. **Assessment of Right Ventricular Function:** It is important in suspected Pulmonary hypertension. Normally, the RV area of the LV area is less than 0.6 the and RV length is less than 0.6 LV length. The dilatation of RV can be graded in the following stages:

*Mild: RV area /LV area = 0.6*

*Moderate: RV area /LV area = 1.0*

*Severe: RV area /LV area >1.0.*

1. **Regional wall motion abnormalities:** Any deviation from the normal contractile pattern is called a regional wall motion abnormality. It can be hypokinetic (segment, which is contracting less than normal), Akinetic (not contracting at all), and Dyskinetic (segment contracting out of synchrony with other segments).


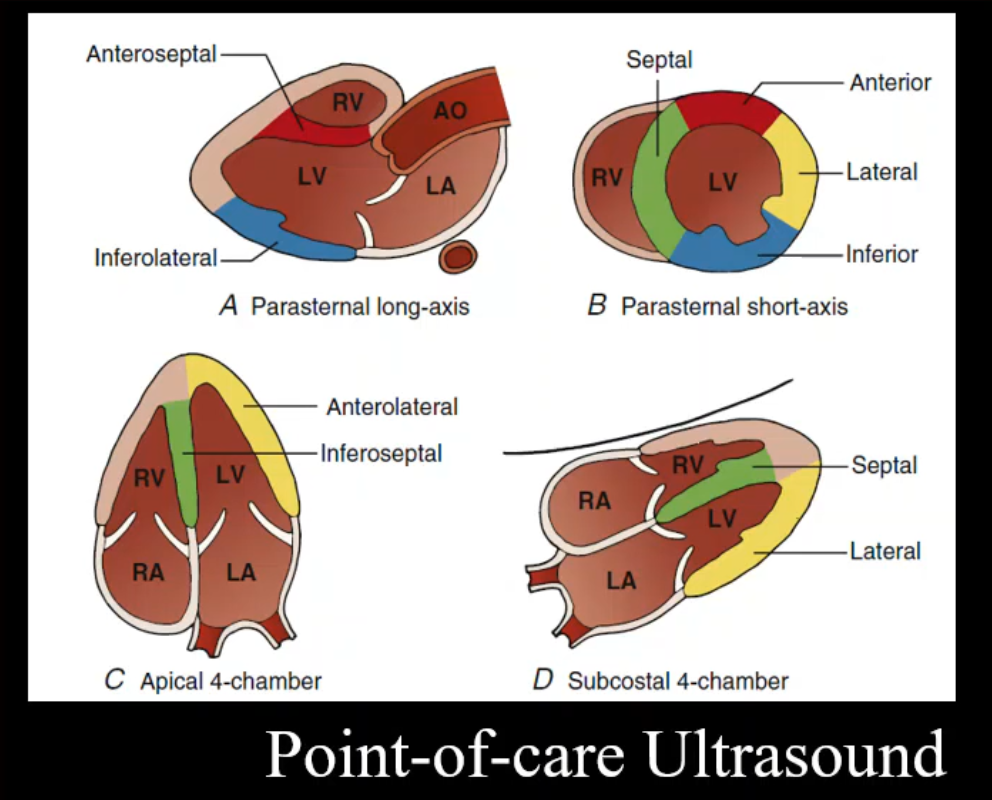


1. **Cardiac tamponade:** Diastolic collapse of RA and RV, more specifically of LA, collection of pericardial fluid around compressed cardiac chambers, dilated IVC with no collapsibility. Apical 4- chamber view and subcostal view are the most useful views.
