# Supplementary material for: Competency of novice anesthesia residents in performing trans thoracic echocardiography following a structured problem-based hands-on course using a trans thoracic echocardiography simulator versus video-based training: a randomized controlled assessor-blinded trial
Source: Adv Simul (Lond). 2026 Jan 24;11:11. doi: 10.1186/s41077-026-00406-1 (PMC12911289; doi:10.1186/s41077-026-00406-1)
Supplement: Supplementary file 3 — Supplementary Material 3. [file 41077_2026_406_MOESM3_ESM.docx]

**Competency score (CS)**

CS is a comprehensive score (0-31) that includes

• Knowledge assessment (MCQ-based): Total (0-10 points)

• Skill assessment (All chamber views, pathological scenarios)

CS is a non-validated score, and validation was done by five experts (both departmental and

interdepartmental), validated by 5 experts from different fields (2 from Anaesthesiology, 1 from Cardiac Anaesthesia, 1 from Cardiology, 1 from simulation lead).

**Validity Evidence (Kane Framework):**

The TTE competency score was developed through expert consensus to evaluate foundational transthoracic echocardiography skill. **Content alignment** was ensured by mapping each scored item to standard TTE training objectives used in perioperative and critical care practice. **Generalisation** was supported by inter-rater reliability during 10 pilot learner examinations (Intraclass correlation coefficient = 0.77, two-way random effects, indicating good agreement). **Extrapolation** was supported by the use of clinically relevant pathological cases reflecting typical perioperative/ICU presentations. The score was used for formative educational assessment, consistent with the **implications** **level** of Kane’s framework, rather than high-stakes certification.

CS=Knowledge assessment (0-10 points) + Skill assessment (5 normal view and 2-pathological scenario) = (0-10) +5(0-3) +2(0-3) =0-31

**Scoring for Normal chamber views: Echo scoring paradigm (0-3) <Total 0-15>**

| **Score** | **Quality of View** | **Description of image** |
| --- | --- | --- |
| 0 | Very Poor | No Image |
| 1 | Poor | <50% of total expected chambers and vessels visualised |
| 2 | Good | > 50% of total chambers and vessels Visualised |
| 3 | Excellent | 100% of total chambers visualised |

**Scoring for Pathological conditions (Critical features of each will be defined) (0-3)**

| **Score** |  |
| --- | --- |
| 0 | Wrong Diagnosis |
| 1 | Description of no critical features |
| 2 | Description of partial critical features |
| 3 | Description of all Critical features |

The endpoint of the assessment was Image plane acquisition of 5 standard TTE views, identifying critical features of each pathology
